# Necrotizing Sarcoid Granulomatosis: A Difficult Diagnosis

**DOI:** 10.7759/cureus.79650

**Published:** 2025-02-25

**Authors:** Carolina Da Silva Alves, Catarina La Cueva Couto, Mariana Silva, Catarina Paulo, Luís Carreto

**Affiliations:** 1 Pulmonology Department, Unidade Local de Saúde Amadora/Sintra, Amadora, PRT; 2 Critical Care Department, Hospital Garcia de Orta and Centro Diagnóstico Pneumológico Administração Regional de Saúde de Lisboa e Vale do Tejo (ARSLVT), Almada, PRT

**Keywords:** granulomas, necrotizing sarcoid granulomatosis, sarcoidosis, systemic inflammatory disease, tuberculosis, vasculitis

## Abstract

Necrotizing sarcoid granulomatosis (NSG) is a rare systemic inflammatory disease characterized by sarcoid-like granulomas, vasculitis, and necrosis.

This case report describes a 40-year-old man from Guinea-Bissau who presented with weight loss, fever, cough, pleuritic chest pain, dyspnea on minimal exertion, and asthenia. Chest computed tomography (CT) revealed a loculated left pleural effusion and lymphadenopathy in the mediastinal, bilateral supraclavicular, and left axillary regions. The initial investigation suggested granulomatous disease with necrosis. Although there was no microbiological evidence of an infectious etiology, pleural and lymph node tuberculosis was presumed due to the patient's origin from a tuberculosis-endemic region. However, the lack of clinical and radiological improvement despite anti-bacillary therapy prompted further investigation. Follow-up imaging revealed a nodular pattern with lymphatic distribution in the left lung, and histopathological analysis of mediastinal lymph nodes and bronchial biopsies showed granulomas with caseous necrosis. Sarcoidosis was considered, but after a multidisciplinary discussion, a final diagnosis of NSG was established. Following the initiation of corticosteroid therapy, the patient experienced significant clinical and functional improvement, culminating in disease resolution.

NSG poses a significant diagnostic challenge due to its varied and nonspecific presentation, often mimicking more prevalent granulomatous diseases. This case underscores the importance of considering NSG in the differential diagnosis of granulomatous diseases, particularly in tuberculosis-endemic regions, and highlights the role of immunosuppressive therapy in achieving disease resolution.

## Introduction

Necrotizing sarcoid granulomatosis (NSG) is an uncommon systemic inflammatory disease characterized by the formation of sarcoid-like granulomas, vasculitis, and necrosis [1-3]. It was first described in 1973 by an American pathologist, Liebow, and since then, only approximately 135 cases have been reported worldwide [3], mainly involving lung parenchyma [4].

The etiology and pathogenesis remain unclear [1,4]. Whether NSG is a separate entity, a variant of sarcoidosis, pulmonary vasculitis, or a hypersensitivity reaction remains unclear [1,3,4]. NSG typically presents with respiratory symptoms such as persistent cough, hemoptysis, dyspnea, and chest pain. Systemic symptoms may also manifest, including fatigue, weight loss, and fever. The diagnosis of NSG results from a combination of clinical evaluation, imaging studies, and histopathological examination of a tissue biopsy, commonly obtained via surgical lung biopsy [1-4]. The hallmark of NSG on histopathological examination is the presence of granulomas with central necrosis. These necrotic areas are surrounded by inflammatory cells, including multinucleated giant cells, lymphocytes, and macrophages [5-10].

NSG shares histological and clinical similarities with several granulomatous conditions, including tuberculosis (TB), environmental and occupational exposures, drug-induced reactions, vasculitides, and sarcoidosis. It is essential to accurately exclude these similar conditions in order to diagnose NSG, as it is ultimately a diagnosis of exclusion. Misdiagnosis could lead to inappropriate treatment and potentially serious clinical outcomes [9].

Due to the rarity of NSG and its unique characteristics, there is no standardized treatment [2]. The clinical course is generally benign, often showing significant improvement with corticosteroid treatment or even spontaneous remission [4,7]. However, the response to treatment can vary among individuals [2,5].

This case report highlights the clinical and diagnostic challenges of NSG, particularly in TB-endemic regions, where its distinction from TB is difficult due to overlapping clinical and histopathological features. This often leads to misdiagnosis and unnecessary anti-TB treatment. A key distinguishing factor in this case is the unilateral involvement, which initially presented with isolated pleural, mediastinal, and supraclavicular lymph node disease before progressing to pulmonary involvement. Additionally, this report explores the therapeutic implications of NSG, emphasizing the critical role of corticosteroid therapy in achieving clinical improvement. By presenting this case, we aim to contribute valuable insights to the growing body of literature on NSG, reinforcing the importance of accurate diagnosis, multidisciplinary evaluation, and recognition of NSG as a differential diagnosis in granulomatous diseases.

## Case presentation

A 40-year-old man from Guinea-Bissau, living in Portugal for 11 years with no significant medical history, history of smoking or drug use, and no record of having received the Bacillus Calmette-Guérin (BCG) vaccine, was admitted to the hospital in February 2022. He was experiencing significant weight loss (10%), fatigue, late afternoon fever, left-sided chest pain, shortness of breath with minimal exertion, and a productive cough with mucoid phlegm for the past six months. The patient denied experiencing night sweats, having a known history of TB contacts, or a personal history of TB.

On physical examination, the patient was febrile (auricular temperature of 38.5ºC) with vital signs showing normotension, a normal heart rate, and a body mass index (BMI) of 20 kg/m². The general inspection revealed an alert and oriented individual with no signs of distress. The skin was unremarkable, with no evidence of erythema nodosum or other dermatological abnormalities. There was no palpable lymphadenopathy in the cervical, axillary, or inguinal regions, and no hepatosplenomegaly was noted. The patient also denied visual changes, and there were no signs of uveitis or conjunctival inflammation. Additionally, the liver was of normal size and consistency, and there were no signs of peripheral edema or clubbing. On respiratory examination, the chest appeared symmetrical, with normal respiratory effort and no use of accessory muscles. However, percussion of the left hemithorax revealed dullness and reduced tactile fremitus, and breath sounds were markedly diminished or absent at the left lung base, suggesting a pleural effusion. A cardiac examination revealed a regular rhythm without murmurs or extra heart sounds, and the abdominal examination was unremarkable. Neurological and musculoskeletal exams were also normal, with no focal deficits or signs of complications.

**Figure 1:**
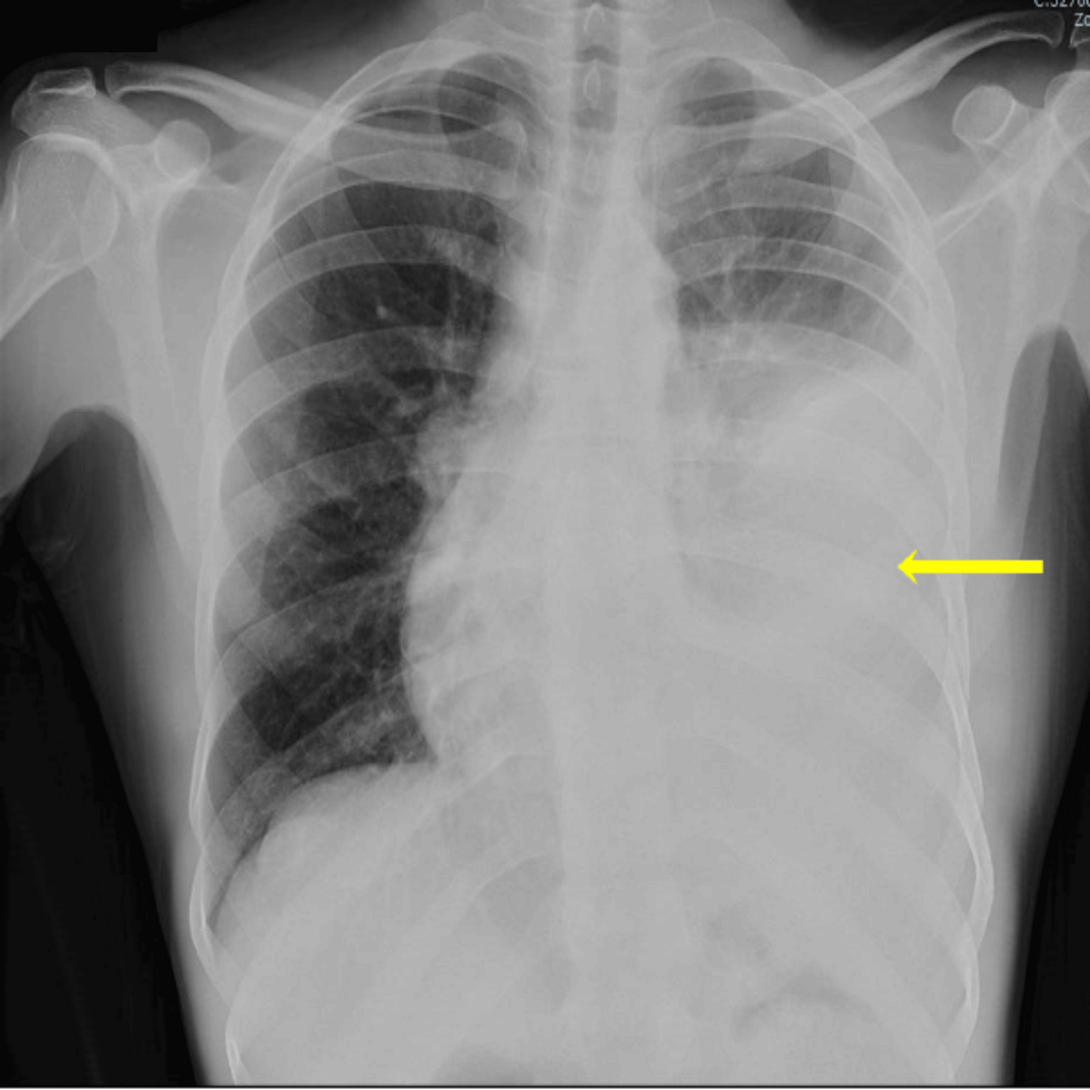
Chest radiograph on hospital admission showing left-sided pleural effusion (arrow).

Laboratory tests at admission showed an erythrocyte sedimentation rate of 81 mm/hour, a leukocyte count of 5.700 with 4.100 neutrophils, and a C-reactive protein (CRP) of 14.1 mg/L. The initial chest radiograph showed left-sided pleural effusion (Figure 1).

**Figure 2:**
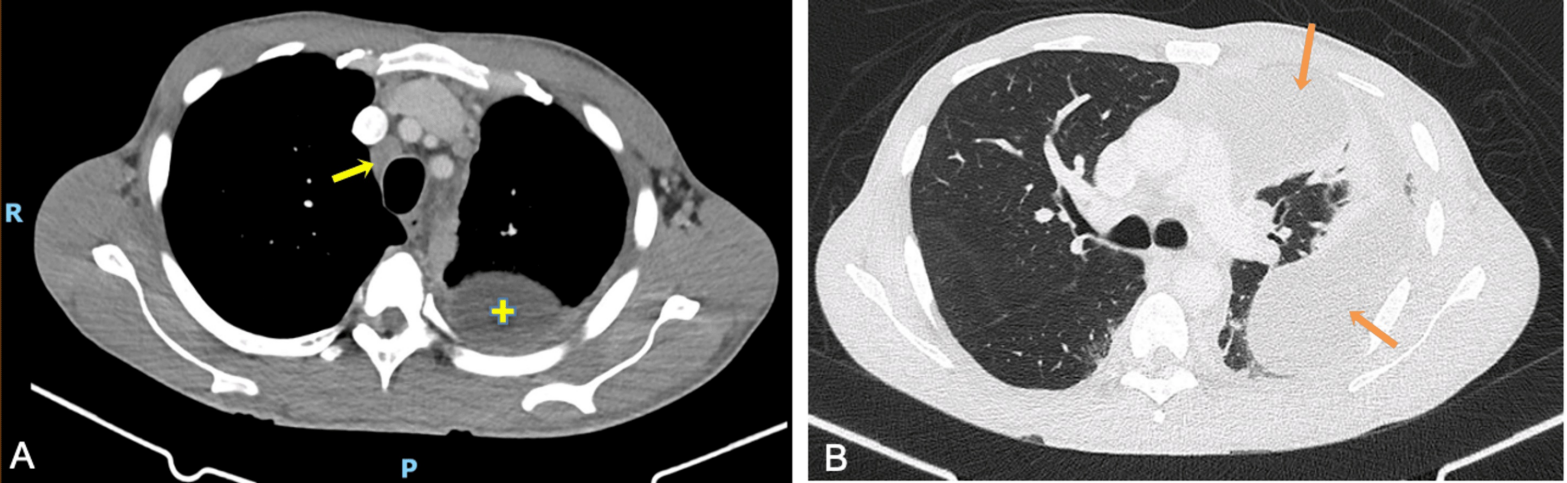
CT scan on hospital admission, transverse plane. Image A shows 4R mediastinal lymphadenopathy (yellow arrow) and an enclosed pleural effusion on the left side (+). Image B, at the level of the carina, highlights the enclosed pleural effusion on the left side (orange arrows).

Subsequent investigation with chest, abdominal, and pelvic CT scans revealed an enclosed pleural effusion on the left side, accompanied by pleural thickening. Additionally, bilateral hilar, mediastinal (4L, 4R, and 7), and supraclavicular lymphadenopathies of varying sizes were identified, as well as lymphadenopathy in the left axillary chain (Figure 2). The largest lymph node measured approximately 20 mm, with the 7 mediastinal lymph node showing signs of necrosis. No parenchymal lesions were observed. A small amount of free fluid was present in the abdominal cavity, but no abdominal or pelvic masses, collections, or other anomalies were noted.

The analysis of the sputum, including smear, culture, and polymerase chain reaction (PCR) by GeneXpert testing, yielded negative results for the *Mycobacterium tuberculosis* complex (MTBC). Thoracentesis revealed an exudative pleural effusion with a predominant lymphocyte count of 89% and adenosine deaminase (ADA) levels of 49 U/L. Cytology of the pleural fluid was negative for malignant cells. A blind pleural biopsy showed granulomatous inflammation with multinucleated giant cells and central necrosis. Smear, culture, and PCR testing for *M. tuberculosis* were negative in both the pleural fluid and pleural biopsy samples.

The patient underwent a flexible bronchoscopy, which identified signs of extrinsic compression of the left basal pyramid and a granular appearance of the left first carina's mucosa. A biopsy of the granular area confirmed caseous necrotizing granulomas, surrounded by Langhans giant cells, with negative cultures and PCR for MTBC. Additionally, bronchoalveolar lavage (BAL) and bronchial secretions yielded negative results for MTBC.

**Figure 3:**
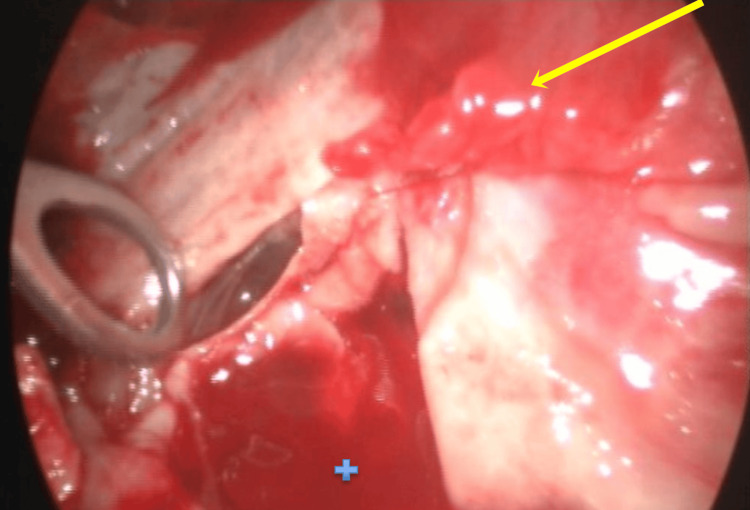
Thoracoscopy showing pleural effusion (+) and a thick pleural adhesion (arrow).

Subsequently, due to the organized pleural effusion and continued diagnostic uncertainty, a medical thoracoscopy was performed, including lysis of pleural adhesions and pleural lavage with saline irrigation (Figure 3). Although pleural biopsies were also performed, no macroscopic alterations in the pleura were identified during the procedure.

The histopathological findings from the pleural samples obtained via thoracoscopy were consistent with those from the blind pleural biopsy. However, persistent loculated pleural effusion and pleural adhesions in the lower half of the left hemithorax were noted. Respiratory rehabilitation was initiated to promote left pulmonary expansion and reduce left pleuropulmonary sequelae.

Autoimmune markers, such as antineutrophil cytoplasmic antibody, were negative.

Given the findings, a diagnosis of TB involving the lymph nodes and pleura was assumed, and anti-TB treatment with isoniazid, rifampicin, pyrazinamide, and ethambutol was initiated. The patient was discharged after one month, with no fever since the start of the treatment, and was followed up at the Center for Pneumological Diagnosis.

**Figure 4:**
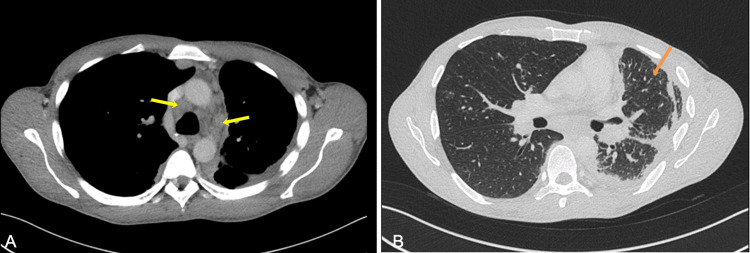
Follow-up chest CT scan after three months of anti-tuberculosis treatment, transverse plane. Image A shows mediastinal lymphadenopathies (4R and 4L) that remained stable despite the treatment (yellow arrows). Image B reveals a new nodular pattern in the left lung with perilymphatic distribution (orange arrow).

After three months of treatment, the patient continued to experience a dry cough, dyspnea during moderate exertion, night sweats, and low body weight. A follow-up chest CT scan showed improvement in the left pleural effusion; however, a new nodular pattern in the left lung with perilymphatic distribution was observed, along with the persistence of lymphadenopathies (Figure 4). Pulmonary function testing identified a mild restrictive ventilatory syndrome, with forced expiratory volume in one second (FEV1) of 2720 mL (66%), forced vital capacity (FVC) of 3210 mL (78%), total lung capacity (TLC) of 5140 mL (77%); and a moderate reduction in carbon monoxide diffusing capacity (DLCO), with corrected single-breath DLCO (DLCOSB) of 6080 mL (58%).

**Figure 5:**
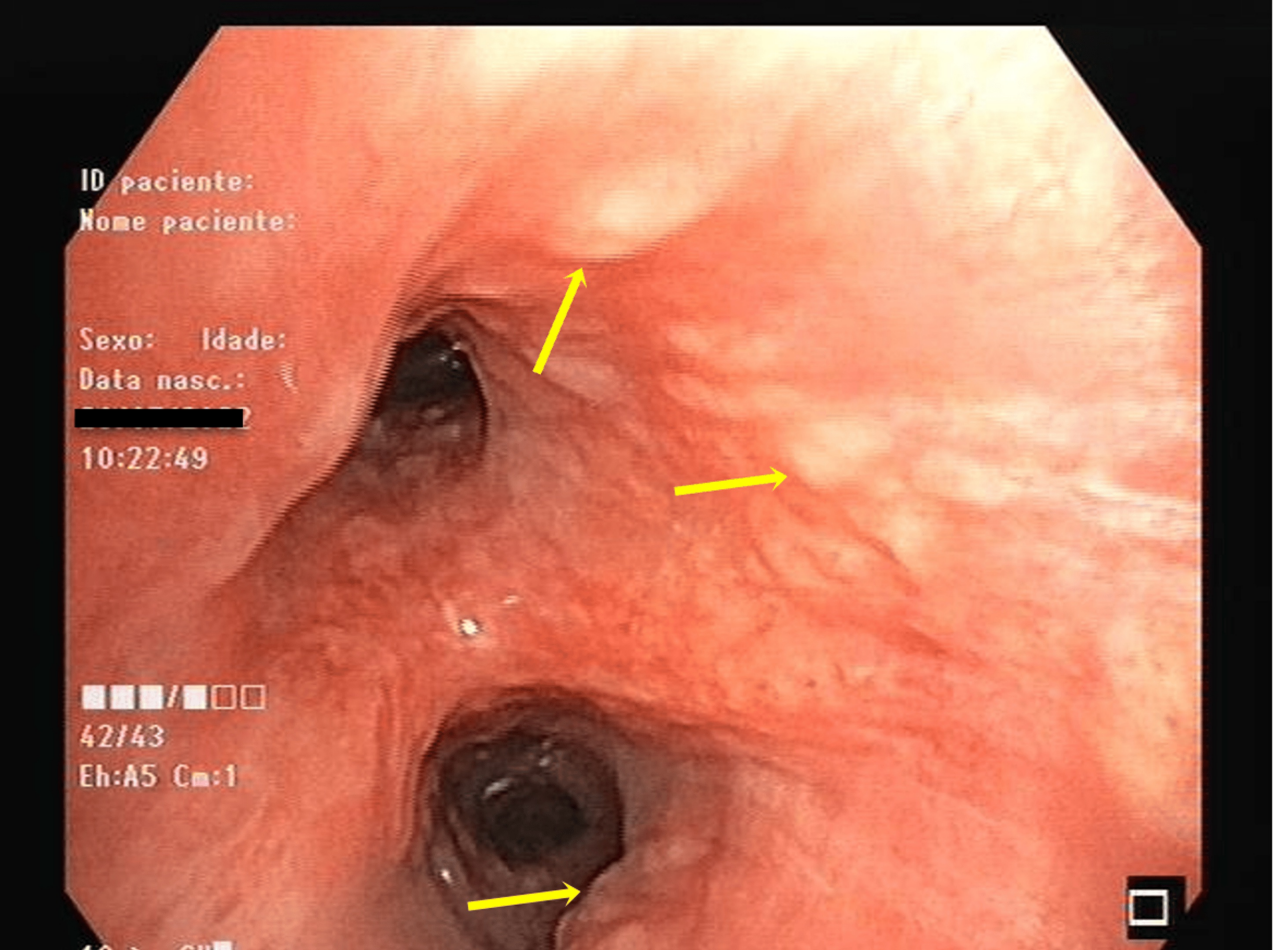
Endobronchial ultrasound bronchoscopy showing granular appearance of the mucosa (arrows).

The patient underwent an endobronchial ultrasound bronchoscopy, which identified a granular appearance of the mucosa (Figure 5) in the main left and right bronchi, as well as six suspicious mediastinal lymph node stations with necrosis (4L, 4R, 11L, 7, 11Ri, 11Rs), of which the last five were biopsied. Histopathological analysis of the biopsied mediastinal lymph nodes and the affected mucosa of the left main bronchus revealed an intense chronic lymphoplasmacytic inflammatory infiltrate with epithelioid granulomas, some displaying caseous necrosis and multinucleated Langhans giant cells. BAL showed lymphocytosis (39%) with a high CD4/CD8 ratio (15.6). Microbiological testing of all three sample types was negative for MTBC.

The previous pleural biopsies were further tested for non-tuberculous mycobacteria and human herpes virus 8 with negative results. Following the exclusion of granulomatous lung infection and primary effusion lymphoma, based on the chest imaging alterations, an angiotensin-converting enzyme (ACE) level of 80 U/L and a high CD4/CD8 ratio in BAL, stage III sarcoidosis (according to Scadding classification) with atypical presentation (involving only the left lung) was suspected. The clinical case was discussed in a multidisciplinary board on interstitial lung diseases, and the diagnosis of NSG was made nine months after the initial presumptive diagnosis.

**Figure 6:**
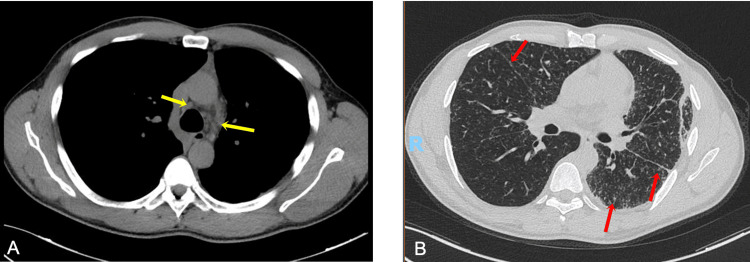
Follow-up chest CT scan after seven months of corticosteroid treatment. Image A shows reduction in the mediastinal lymphadenopathies (yellow arrows). Image B shows improvement of the pleural thickening (yellow arrow) and persistence of the nodular pattern with perilymphatic distribution predominantly in the left lung (red arrows).

Prednisolone was initiated at a dose of 50 mg/day (1 mg/kg/day), and anti-tuberculous treatment was discontinued after nine months. The patient maintained this dose of prednisolone for three months. Due to clinical improvement, a gradual reduction of 5 mg was attempted for three weeks with success. Every three weeks, the dose was reduced by 5 mg. When the dose reached 5 mg/day, it was maintained for one month, during which a new radiologic and functional evaluation was performed. During this follow-up, the patient showed clinical improvement with resolution of the cough and dyspnea. Functionally, there was also improvement, as demonstrated by normal respiratory function tests, with FEV1 of 3150 mL (87.7%), FVC of 3680 mL (83.3%), TLC of 5570 mL (84%), and a mild decrease in DLCOSB to 7350 mL (71%). Radiologic improvement was observed (Figure 6), including a reduction in mediastinal lymphadenopathies and resolution of pleural effusion with pulmonary expansion. However, the nodular pattern with perilymphatic distribution persisted, albeit with less prominence.

Prednisolone was discontinued after a total of seven months of therapy. The patient remains asymptomatic at the most recent evaluation, 17 months after therapy was suspended.

## Discussion

NSG is a rare disease characterized by granulomatous inflammation with necrosis, posing diagnostic challenges often resulting in delayed diagnosis [2,11]. NSG typically presents at an average age of 42 years, predominantly in females, primarily affecting the lungs. There is also variable prevalence of extrapulmonary involvement, which may include the eyes, skin, and nervous system [2,8,12]. Symptoms are often non-specific, and radiologic findings can vary, necessitating reliance on pathological findings for accurate diagnosis [2,9,11]. It primarily affects the lungs, as demonstrated in the presented case. Karpathiou et al. reviewed the clinical, radiologic, and histopathologic findings of NSG, concluding that the most common radiologic presentation is a pattern of multiple lung nodules followed by a solitary nodule or mass [12]. Consequently, distinguishing NSG from malignancy is crucial [13]. Data regarding the prevalence of mediastinal and hilar lymphadenopathies and pleural effusion in NSG are inconsistent across studies [2,4,9].

Histologically, extensive areas of necrosis are observed within the lung parenchyma, which is associated with the occlusion of pulmonary vessels by granulomatous inflammation. The precise etiology remains elusive, particularly whether the sarcoid-like reaction in NSG results from necrotizing vasculitis or represents a distinct process leading to granuloma necrosis, differentiating it from classic sarcoidosis [13].

Vasculitis linked with granulomatous lung disease can manifest in various conditions, encompassing granulomatosis with polyangiitis, nodular sarcoidosis, and infections such as TB or schistosomiasis, as well as foreign body reactions, including pulmonary embolism due to foreign bodies in drug users [2,8,10,13]. Notably, substantial overlap exists between nodular sarcoidosis and the pattern observed in NSG in terms of clinical symptoms and extrapulmonary manifestations, prompting ongoing debate regarding whether NSG represents a distinct entity or merely a variant of sarcoidosis characterized by extensive infarct-like necrosis [14]. The differential diagnosis can be difficult, as each condition requires a distinct therapeutic approach, making accurate diagnosis crucial [8,12]. Unlike other infections or malignancies, a favorable response is typically anticipated with systemic corticosteroid therapy, which is the most utilized treatment for NSG [13]. In contrast, TB necessitates specific TB treatment. Therefore, a lack of microbiological isolation in the samples should lead us to suspect a different granulomatous disease, such as NSG [10].

In the presented case, despite the absence of microbiological evidence of TB, the patient’s constellation of symptoms and radiological findings, necrotizing granulomatous inflammation in the pleural biopsy, high ADA levels in the pleural fluid, and granular appearance of the mucosa of the left main bronchus, combined with the patient’s history of living in an endemic region (Guinea-Bissau), made pleural and lymph node TB the most probable diagnosis, warranting treatment according to the guidelines on treatment of TB [14]. As highlighted by Chong et al., NSG can often be mistaken for granulomatous infections like TB, particularly in endemic regions [8]. This resemblance further complicated the diagnostic process in this case.

The patient remained symptomatic with dry cough, dyspnea, night sweats, and low weight after three months of anti-TB treatment. On the follow-up chest CT, the nodular pattern with lymphatic distribution in the left lung was consistent with sarcoidosis; however, sarcoidosis is typically bilateral and does not usually present with pleural effusion [2]. Interestingly, ACE concentration was elevated in this patient, as in 60% of patients with active sarcoidosis [2]. Usually, ACE concentration is normal in NSG [2,10]. It should be noted that the presence of intrathoracic lymphadenopathies is more frequent in sarcoidosis than in NSG [2,8]. Also, other histological features typical of pulmonary sarcoidosis (non-necrotizing confluent granulomas surrounded by hyaline fibrosis and necrosis) and granulomatosis with polyangiitis (geographical areas of basophilic necrosis) are frequently unseen in NSG, as in this case [9].

Nonetheless, the consistent presence of necrotizing granulomas in the biopsy specimens, along with negative TB-specific tests, no response to TB treatment after nine months, and exclusion of other infectious agents such as non-tuberculous mycobacteria, prompted further consideration of NSG as a possible diagnosis. Typically, tissue obtained by surgical procedures is necessary for histopathological analysis, especially when less invasive tests are inconclusive. In this case, the patient had mediastinal and hilar adenopathies that were easily accessible through endobronchial ultrasound bronchoscopy, as well as subsequent changes in the bronchial mucosa that were biopsied. Additionally, the patient underwent thoracoscopic pleural biopsy, which has a diagnostic yield as high as 100% for pleural TB.

Similar to the clinical case presented, Parejo-Morón et al. described two cases of NSG initially misdiagnosed as TB, for which anti-bacillary treatment was initiated. Due to the lack of a favorable response, a biopsy was performed, leading to the correct diagnosis and allowing corticosteroid treatment to be initiated with a good response [11].

The prognosis for NSG patients is typically favorable [2,10], as evidenced by this case. Additionally, spontaneous regression of the disease has been documented [2,13]. However, one case of severe neural involvement leading to fatality has been reported [3]. NSG is sensitive to corticosteroid therapy, with treatment typically involving daily administration of prednisone at doses ranging from 40 to 60 mg for a duration of four to eight weeks [13]. Other forms of immunosuppression, such as methotrexate, have also been reported [11,13].

## Conclusions

This case of NSG in a 40-year-old man showcases unique clinical and radiological features that deviated from typical presentations of granulomatous diseases. Initially suspected to be pleural and ganglionic TB due to the patient's symptoms and endemic background, a conclusive diagnosis of NSG was only established through extensive multidisciplinary evaluation, including histopathological analysis and the exclusion of other potential diseases such as primary effusion lymphoma and various infections. The resolution of symptoms and significant improvement in clinical, functional, and radiological parameters following corticosteroid therapy underline the importance of considering NSG in differential diagnosis.

This case highlights the critical role of comprehensive diagnostic processes in managing atypical granulomatous disorders and underscores the effectiveness of corticosteroid therapy in achieving favorable outcomes. Furthermore, this case report emphasizes the necessity of considering a broader spectrum of granulomatous diseases, including NSG, particularly in patients presenting with atypical symptoms and findings. This approach can guide more accurate and effective clinical management.
